# A non-persistent aphid-transmitted *Potyvirus* differentially alters the vector and non-vector biology through host plant quality manipulation

**DOI:** 10.1038/s41598-019-39256-5

**Published:** 2019-02-21

**Authors:** Kiran R. Gadhave, Bhabesh Dutta, Timothy Coolong, Rajagopalbabu Srinivasan

**Affiliations:** 1 Department of Entomology, University of Georgia, 1109 Experiment Street, Griffin, GA 30223 USA; 2 Department of Plant Pathology, University of Georgia, 2360 Rainwater Road, Tifton, GA 31793 USA; 3 Department of Entomology and Plant Pathology, North Carolina State University, Raleigh, NC 27695 USA

## Abstract

The association of plant viruses with their vectors has significant implications for virus transmission and spread. Only a few studies, with even fewer pathosystems, have explored non-persistent (NP) virus-vector interactions that are presumed to be transient. We studied how a NP virus, *Papaya ringspot virus* (PRSV) influenced the behavior and biology of its vector, the melon aphid (*Aphis gossypii* Glover) and the non-vector, silverleaf whitefly (*Bemisia tabaci* Gennadius). We also assessed whether the fitness effects on aphids are modulated through changes in the host plant, squash (*Cucurbita pepo* L.) nutrient profile. The overall performance of *A. gossypii* was substantially higher on PRSV-infected plants, along with increased arrestment on PRSV-infected than non-infected plants. No such PRSV-modulated fitness effects were observed with *B. tabaci*. PRSV-infected plants had increased concentrations of free essential amino acids: threonine, arginine and lysine; non-essential amino acids: glycine and homocysteine; and soluble carbohydrates: galactose, raffinose and cellobiose. In general, PRSV encouraged long-term feeding and enhanced fitness of *A. gossypii* through host plant nutrient enrichment. These findings provide evidence for a NP virus mediated positive fitness effects on its vector, with no spillover fitness benefits to the non-vector within the same feeding guild.

## Introduction

A majority of plant viruses rely on insect vectors for plant-to-plant dispersal. Plant viruses transmitted in a persistent manner are known to be strongly associated with insect vectors. An array of these viruses manipulates vector behavior and biology in ways that favor their spread to non-infected plants^1–4^. The association of non-persistent (NP) viruses with their vectors appear to be transient and/or context-specific, resulting in variable effects ranging from increased attraction to infected plants and rapid dispersal to preferred alightment and arrestment on infected plants and enhanced fitness^5–7^. For instance, *Cucumber mosaic virus* (CMV), a widely studied non-persistent *Cucumovirus* (Family *Bromoviridae*), has been reported to enhance aphid attraction to infected cucurbit plants, and encourage rapid aphid dispersal following virus acquisition through increased host plant volatile emissions and reduced host plant quality^8^. Conversely, other aphid-transmitted non-persistent viruses, including *Potato virus Y* (PVY), *Turnip mosaic virus* (TuMV) and *Zucchini yellow mosaic virus* (ZYMV) have been reported to counter plant host defenses and/or enrich host plant nutritional quality for aphids^9–13^. When compared with persistent viruses, fewer studies have examined NP pathosystems. Therefore, whether and how component interactions identified in select NP pathosystems are prevalent across other important yet unexplored NP pathosystems remains to be seen.

Vector-virus interactions may be influenced through a variety of virus, plant, and insect factors^7^ *viz*. viral proteins^12–14^, plant volatile profiles and nutrient status^15^, plant constitutive and induced defenses^16^, and insect response to visual, sensory and olfactory cues^1,15,17^. Some of the visual, sensory and olfactory cues may modify vector behavior on a short-term basis, as they mostly trigger vector attraction and preference. Visual cues emanating from virus-infected plants such as yellowing seem to make the plants more apparent to their vectors^18–20^. The virus-induced changes in plant volatile profiles also appear to affect vector behavior in a context-specific manner, with some studies showing positive manipulation^1,4,21^, while the others showing no manipulation^22^. Once the vectors are settled, host plant quality could play a critical role in determining long-term effects of virus on vectors^7^.

It is proposed that the effects of viruses on overall host plant quality are dependent on the mode of transmission^2,5^. Although this appears to be true, in most part, with persistent viruses, the literature on NP virus-vector interactions does not indicate a generic pattern. Studies on NP viruses have reported favorable, neutral, and detrimental effects on host plant quality^5^. Each of these effects appear to have specific consequences on long-term settling and development of vectors^5,15,23^. It was recently hypothesized that variation in (i) degree of virus adaption to host, (ii) effects of co-occurring hosts- with some viruses promoting and others discouraging vector settling, and (iii) vector behavior and population density contribute to variation in NP virus modulated effects on vector-plant interactions^6^. While conventional hypothesis highlights the detrimental effects of long-term settling of vectors on NP virus spread, more recently a contradicting hypothesis was proposed^6^. It states that long-term settling of NP virus vectors could encourage virus spread by increasing vector population density and early alate development. The speculation is that the long-term settling and subsequent development of alatae would foster the establishment of large number of inoculum foci over greater distances than the rapid and fewer inoculum foci due to quick vector dispersal^6^. Most of these virus driven plant and vector manipulations are likely mediated through biochemical determinants such as free amino acids, soluble carbohydrates, and defense signaling compounds^5–7^. Free amino acids and soluble carbohydrates especially seem to be key contributors to host plant quality for vectors. Virus mediated changes in their profiles have been reported in earlier studies^24–26^. These changes have been reported to influence aphid biology in both a positive^27–29^ and negative manner^5,30^. To date, almost no empirical work has explored whether and how NP virus manipulates these primary plant metabolites to offer selective fitness benefits to vector over non-vector insects.

*Papaya ringspot virus*, a single-stranded positive sense RNA *Potyvirus*, is primarily grouped into papaya infecting type (PRSV-P) and the cucurbit infecting type (PRSV-W)^31^. The former strain infects both papaya and cucurbits, while the latter one infects only cucurbits. PRSV-W strain and other major potyviruses cause substantial cucurbit production losses worldwide. PRSV-W (referred hereafter as ‘PRSV’) is transmitted by several species of aphids in a non-persistent manner. Melon aphid (*Aphis gossypii* Glover) is the most efficient vector of this virus^32^, and is an economically important pest of cucurbits throughout the world. Apart from examining PRSV transmission efficiency of *A. gossypii*, *Aphis craccivora* (Koch), and *Myzus persicae* (Sulzer)^32^, vector-virus interactions in a cucurbit-PRSV- aphid pathosystem remain unexplored. Furthermore, it is not clear whether the virus-mediated effects on vectors are specific or if they would also impact non-vectors that belong to the same feeding guild as the vector and are simultaneously exposed to virus-mediated changes in the host plant. To explore such indirect effects, we examined the effects of PRSV-infection on silverleaf whitefly, *Bemisia tabaci* (Gennadius), a ubiquitous non-vector species sharing the same phloem feeding guild along with the vector in the current study system.

We examined the effects of PRSV infection in squash on the preference and biology of its vector; *A. gossypii* and a ubiquitous non-vector; *B. tabaci*. We further studied the effects of PRSV on squash free amino acid and soluble carbohydrate profiles. In line with the consensus on vector-virus interactions, we hypothesized that PRSV infection in squash will increase attractiveness of *A. gossypii*, will modify host nutrient profiles drastically, and consequently provide long term fitness benefits to the vector. These PSRV-induced changes in squash, however, may not influence the performance of non-vector whiteflies on those plants, mostly due to the lack of virus-driven selection pressure, with no apparent evolutionary benefit to non-vector.

## Results

### Aphid and whitefly response to PRSV-infected and non-infected leaves

Dual-choice settling bioassays were conducted to determine the aphid and whitefly settling preferences to PRSV-infected versus non-infected squash plants. The aphid *A. gossypii* adults did not show any robust preference to either PRSV-infected or non-infected leaves (F_(1,38)_ = 1.43, P = 0.23). Only ~25 of the 100 aphids in each trial settled on either of the leaves. The remaining aphids did not show any settling preference. The whitefly *B. tabaci* settling also followed a similar pattern. There was no clear preference to either of the leaves (F_(1,38)_ = 1.45, P = 0.23). Unlike aphids, the majority of whiteflies (approx. 67%) settled on one of the two leaves.

### Aphid emigration from and immigration to PRSV-infected and non-infected leaves

**Figure 1:**
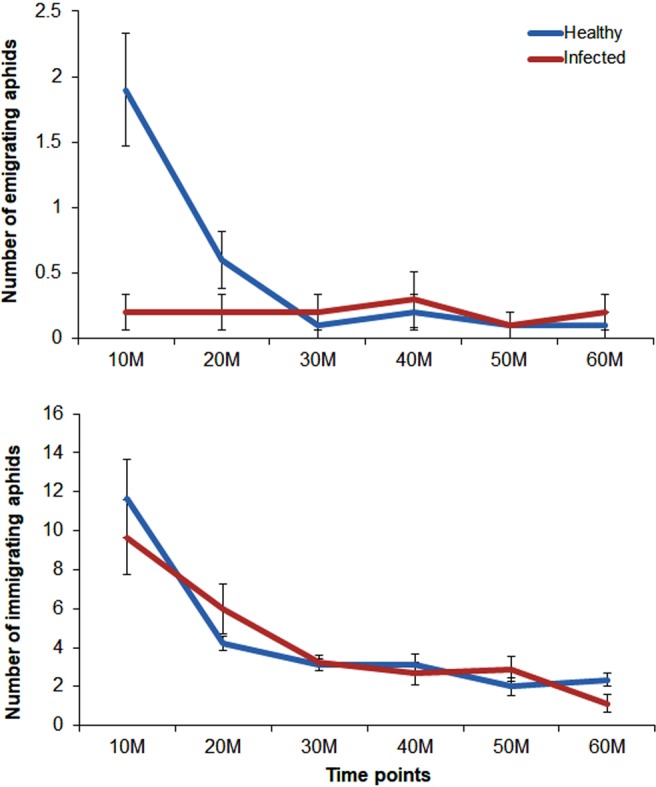
Number of emigrating and immigrating aphids on PRSV-infected and non-infected plants at 10 min time intervals (Mean ± SEM, n = 10).

The movement of *A. gossypii* away from and towards PRSV-infected versus non-infected plants was studied through emigration and immigration assays, respectively. Continuous observations of aphids at every 10 min for 1 h showed higher numbers of individuals emigrating from non-infected plants, when compared with the PRSV-infected plants at the first two time points (Fig. 1). As a result, a significant treatment (Z_(1,38)_ = −3.84, P = 0.0001), time (Z_(1,38)_ = −4.76, P < 0.0001) and treatment: time interactions (Z_(1,38)_ = 3.23, P = 0.0012) were observed. The aphid numbers beyond the third time point; however, were nearly similar.

On the contrary, numbers of aphids immigrating towards both infected and non-infected leaves were nearly similar at every time point (Fig. 1). Furthermore, these numbers gradually declined at every time point, with highest numbers at the first time point (Non-infected = 12; Infected = 10) and the lowest at the last one (Non-infected = 2; Infected = 1). Thus, no significant effects of treatment (Z_(1,38)_ = −0.093, P = 0.92) and treatment: time interactions (Z_(1,38)_ = −0.11, P = 0.90) were observed. The time term (Z_(1,38)_ = −4.76, P < 0.0001); however, was significantly different suggesting significant variation in mean number of aphids immigrating at different time points.

### *A. gossypii* and *B. tabaci* life history traits

**Figure 2:**
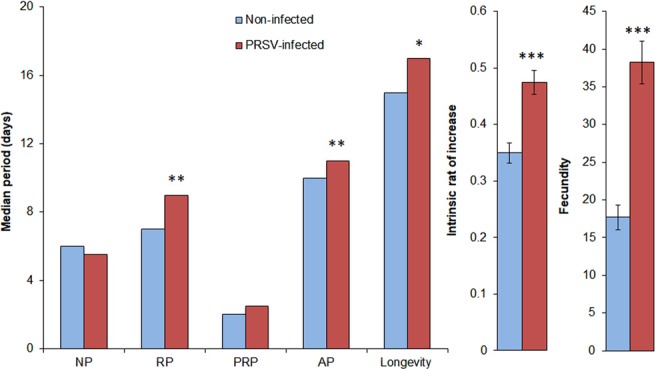
Life history traits of *A. gossypii* on PRSV-infected and non-infected plants. NP = Nymphal period, RP = reproductive period, PRP = Pre-reproductive period, AP = adult period. The time period values are represented in median (n = 20). Asterisks indicate significant effects (*P ≤ 0.05, **P ≤ 0.01, ***P ≤ 0.001, by Mann-Whitney *U* Test/ANOVA).

We determined the long-term effects of PRSV on vector and non-vector insects through comparatively analyzing their life history traits on virus-infected versus non-infected plants. The median *A. gossypii* reproductive period was significantly longer on PRSV-infected plants than on non-infected plants (RP: W_(1,38)_ = 93, p = 0.006) (Table 1) (Fig. 2). Similarly, adult period was significantly extended on infected than on non-infected plants (W_(1,38)_ = 82.5, p = 0.0024). As a result, longevity was also higher on infected than on non-infected plants (W_(1,38)_ = 125, P = 0.025). However, no statistical differences were observed in the nymphal periods of *A. gossypii* developed on non-infected and PRSV-infected plants (W_(1,38)_ = 257.5; P = 0.19). Showing consistent patterns with RP, AP and longevity, fecundity and intrinsic rate of increase were substantially higher on infected as opposed to non-infected plants (fecundity: F = 41.4, P < 0.001; intrinsic rate: F_(1,38)_ = 20.3, P < 0.001) (Fig. 2). Median development time (egg-adult) of *B. tabaci* did not vary between infected and non-infected plants (20 d in each) (W_(1,38)_ = 275, P = 0.49). Mean fecundity on infected plants was not different from non-infected plants (F_(1,38)_ = 1.3, P = 0.26) (Table 1).

**Table 1:** Life history traits of (a) *A. gossypii* and (b) *B. tabaci* on PRSV-infected and non-infected plants.

Life history traits		
Mann-Whitney *U* Test	W	P
**(a)** ***A. gossypii***		
1 Nymphal Period	257.5	0.19
**2 Reproductive Period**	**93**	**0.006**
**3 Adult Period**	**82.5**	**0.0024**
**4 Longevity**	**125**	**0.025**
**One-way ANOVA**	**F**	**P**
**5 Fecundity**	**41.49**	**<0.0001**
**6 Intrinsic rate**	**20.34**	**<0.0001**
**(b)** ***B. tabaci***		
**Mann-Whitney** ***U*** **Test**	**W**	**P**
1 Median developmental time	275	0.49
**One-way ANOVA**	**F**	**P**
2 Fecundity	1.3	0.26

Significant effects (*P* ≤ 0.05 by Mann-Whitney *U* Test/ANOVA) are specified in bold.

### Soluble carbohydrate and free amino acid analyses

**Figure 3:**
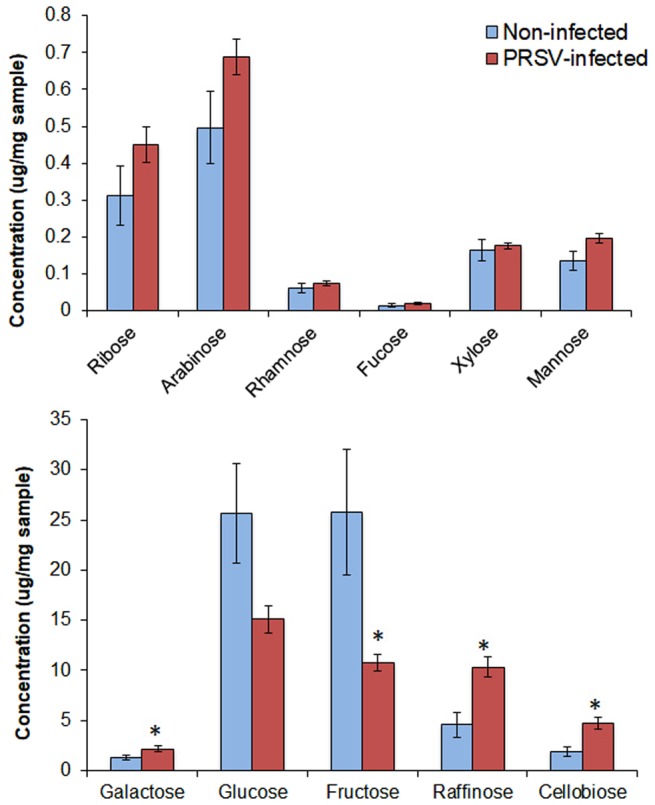
Soluble carbohydrate analysis of PRSV-infected and non-infected plants (Mean ± SEM, n = 5). Asterisks indicate significant effects (*P ≤ 0.05, **P ≤ 0.01, ***P ≤ 0.001, by Mann-Whitney *U* Test).

To assess whether the effects of PRSV on the life history traits of *A. gossypii* were mediated through changes in squash nutrient profiles, we analyzed the qualitative and quantitative changes in soluble carbohydrates and free amino acids of squash. Of the 11 mono- and di- saccharides analyzed, four were significantly different between PRSV-infected and non-infected plants (n = 5) (Fig. 3). The concentrations of galactose (W_(1,8)_ = 2, P = 0.031), raffinose (W_(1,8)_ = 1, P = 0.021) and cellobiose (W_(1,8)_ = 2, P = 0.036) were significantly higher in PRSV-infected plants (Table 2). On the contrary, the concentrations of two predominant sugars, glucose (W_(1,8)_ = 19, P = 0.20) and fructose (W_(1,8)_ = 23, P = 0.031), were significantly higher in non-infected plants than infected plants. Overall, total carbohydrate concentrations were not significantly different between infected and non-infected plants (W_(1,8)_ = 16, P = 0.54).

**Table 2:** Analysis of (a) soluble carbohydrates and (b) free amino acids in PRSV-infected and non-infected plants.

	W	P
(a) Carbohydrates		
1 Ribose	**5**	0.15
2 Arabinose	**5**	0.15
3 Rhamnose	**7**	0.28
4 Fucose	6.5	0.21
5 Xylose	6.5	0.21
6 Mannose	5	0.11
**8 Galactose**	**2**	**0.031**
10 Glucose	19	0.20
**11 Fructose**	**23**	**0.031**
**12 Raffinose**	**1**	**0.021**
**13 Cellobiose**	**2**	**0.036**
Total	16	0.54
**(b) Amino acids**		
1 Taurine	18	0.30
2 Aspartic acid	20	0.15
**3 Threonine**	**1**	**0.015**
4 Serine	5	0.15
5 Glutamic acid	21	0.095
6 Glutamine	10	0.69
7 Sarcosine	3	1
**8 Glycine**	**1**	**0.016**
9 Alanine	15	0.69
10 Citrulline	5	0.15
11 Valine	12	1
12 Methionine	5	0.15
13 Isoleucine	12	1
14 Leucine	12	1
15 Phenylalanine	12	1
16 b-alanine	12	1
17 g-aminobutyric acid	21	0.095
**18 Homocystine**	**0**	**0.007**
19 Ethanolamine	19	0.22
20 Ammonia	8	0.42
**21 Lysine**	**0**	**0**.**028**
22 Histidine	6	0.22
23 Carnosine	1	1
**24 Arginine**	**0**	**0.028**
25 Hydroxyproline	5	0.8
26 Asparagine	11	0.83
**27 Proline**	**23**	**0.031**
28 a-aminobutyric acid	2	1
39 Tyrosine	9	0.9
30 Tryptophan	2	0.14
Total	10	0.69

Significant effects (*P* ≤ 0.05 by Mann-Whitney *U* Test) are specified in bold.

**Figure 4:**
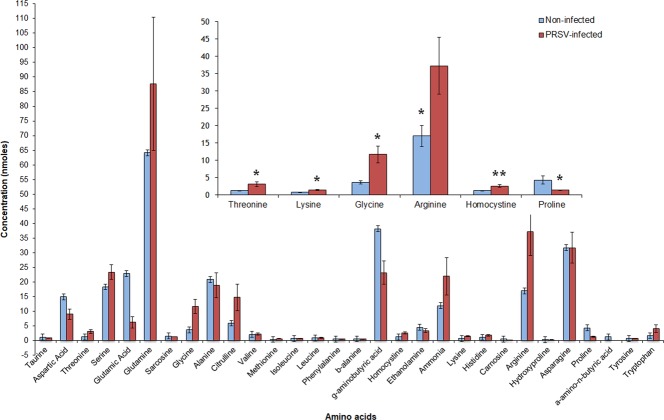
Free amino acid analysis of PRSV-infected and non-infected plants (Mean ± SEM, n = 5). Asterisks indicate significant effects (*P ≤ 0.05, **P ≤ 0.01, ***P ≤ 0.001, by Mann-Whitney *U* Test).

**Figure 5:**
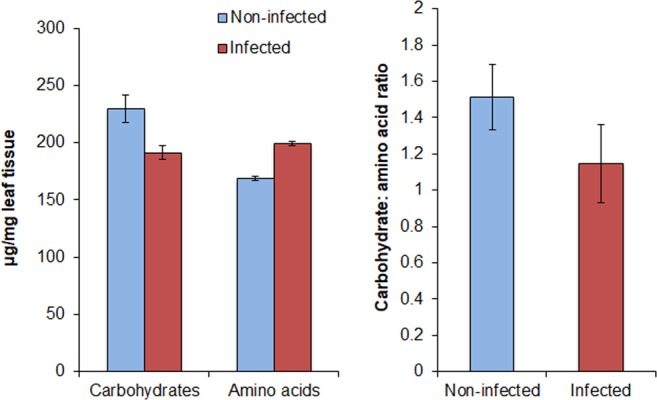
Overall nutrient status of PRSV-infected and non-infected plants (Mean ± SEM).

Six of the 30 free amino acids were significantly different between PRSV-infected and non-infected plants (*n* = 5) (Fig. 4). Three essential amino acids; threonine (W_(1,8)_ = 1, P = 0.015), lysine (W_(1,8)_ = 0, P = 0.028) and arginine (W_(1,8)_ = 0, P = 0.028) and two non-essential amino acids; glycine (W_(1,8)_ = 1, P = 0.016) and homocysteine (W_(1,8)_ = 0, P = 0.007) were significantly higher in PRSV-infected plants than non-infected plants (Table 2). On the contrary, the non-essential amino acid; proline (W_(1,8)_ = 23, P = 0.031) was significantly lower in PRSV-infected plants. Like carbohydrates, total free amino acids concentrations were not significantly different between these two treatments (W_(1,8)_ = 10, P = 0.69). The ratio of soluble carbohydrates to free amino acids was not significantly different between non-infected and PRSV-infected plants (W_(1,8)_ = 20, P = 0.15) (Fig. 5).

## Discussion

We studied whether PRSV impacts the preference and fitness of a vector and a non-vector of the same feeding guild in a cucurbit pathosystem. Existing literature on select NP viruses highlights their inconsistent interactions with vectors. By exploring an economically important yet sparsely explored pathosystem, the present study demonstrated NP virus-mediated arrestment and long-term fitness benefits to its insect vector. These benefits can be evidenced by increased *A. gossypii* performance on PRSV-infected plants, in which the concentrations of five amino acids and three carbohydrates that are necessary to the nutrition and development of *A. gossypii* were elevated. Meanwhile, *B. tabaci* preference and development were not significantly altered on PRSV-infected squash. This was presumably due to very specific effects of PRSV-mediated nutritional manipulation on vectors, with no spill over effects to take advantage by *B. tabaci* although it belonged to the same phloem feeding guild as *A. gossypii*.

For both *A. gossypii* and *B. tabaci*, we did not find any significant settling differences between PRSV-infected and non-infected plants. Similar results have been presented previously in three studies with a NP virus, PVY, in which no settling responses of *M. persicae* and *Macrosiphum euphorbiae* (Thomas) were observed^1,33,34^. Results from the present study differed from those of similar studies in which CMV infection in squash^15^ and in tobacco^35^, ZYMV infection in squash^11^, and TuMV infection in *Nicotiana benthamiana*^12,13^ increased the attraction and/or settling of *M. persicae* and/or *A. gossypii* to their respective infected host plants. Phloem feeding insects are known to gauge plant quality information from gustatory cues available during initial probes^8^. Although the probing behavior may vary from one aphid species to the other for the same virus^10^ and from one pathosystem to the other^36^. Since phloem-feeders principally use sucrose to locate phloem^37^, its natural low concentrations in squash may have made it difficult for *A. gossypii* and *B. tabaci* to prefer either of the plants in a robust way and may have contributed to poor overall settling of aphids (~25%).

Number of *A. gossypii* emigrating from non-infected plants was significantly higher at the first 10 min time interval and decreased subsequently at second- and third-time intervals. However, the actual numbers of emigrating aphids were substantially low in both treatments. On the contrary, these numbers were much higher for aphids immigrating towards both plants; however, no significant differences were observed. The minimal movement of aphids away from PRSV-infected plants show similarity with a few earlier studies^11–13^, while disparity with others^15,22^. In the latter, the aphid vectors *M. persicae* and *A. gossypii* readily emigrated from CMV-infected squash^15^, similarly *Rhopalosiphum maidis* (Fitch) readily emigrated from *Soybean mosaic virus* (SMV)-infected soybean^22^. *M. persicae* emigration; however, was not significantly different from PVY-infected potato over untreated control^1^. Soluble carbohydrates could partly explain the movement of *A. gossypii* towards and away from PRSV-infected plants. Earlier study by Hewer *et al*.^37^ showed that in the absence of sucrose in leaves, raffinose strongly stimulates aphid probing. Since the concentrations of raffinose were significantly higher in PRSV-infected plants in the absence of sucrose, it is possible that *A. gossypii* responded to raffinose stimulated probing in the first 10–20 minutes^38^. As a result, no aphids emigrated from PRSV-infected plants, while relatively more emigrated from non-infected plants. Based on the first probing, aphids are either stimulated to initiate vascular feeding or to disperse^38^. The *A. gossypii* feeding appeared to be stimulated on PRSV-infected plants, but not on non-infected plants.

PRSV mediated changes in host plant quality influenced the performance of *A. gossypii*. Significant increase in the reproductive potential of *A. gossypii*, as evidenced by longer reproductive and adult periods and longevity, and increased fecundity and intrinsic rate appear to be due to profound increase in essential amino acids: threonine, arginine and lysine, and soluble sugars: galactose, raffinose and cellobiose in PRSV-infected plants. An array of previous studies has highlighted the roles of essential amino acids and soluble carbohydrates in elevating the performance of aphids^39–46^. Threonine, arginine and lysine, being essential amino acids, are critical constituents of aphid haemolymph, saliva and honeydew^39,46,47^. Furthermore, all three amino acids have been shown to positively influence aphid performance^39,46^. Glycine and homocysteine, although classified as non-essentials, when supplemented along with essential amino acids, increased aphid performance^48^. Furthermore, these and other non-essential amino acids are utilized by aphid endosymbionts as precursors to synthesize essential amino acids^45,46,49^.

Aphids principally use glucose, fructose, sucrose, galactose and raffinose to meet their dietary requirements^41,49–51^. Consistent with multiple previous studies in *Cucurbita* genus^8,52^, we found sucrose in traces in both infected and non-infected squash plants and therefore its concentrations have not been reported. In the absence of sucrose, fructose and glucose were most abundant phloem sugars in both non-infected and PRSV-infected plants. In the present study, raffinose has been the most abundant sugar following fructose and glucose and its substantial increase in PRSV-infected plants, along with essential amino acids and sugars, may likely have encouraged *A. gossypii* growth. In a diet deficient in sucrose, raffinose has been reported to be critically important sugar that supports aphid growth^41^. A carbohydrate to amino acid ratio below 8:1 has been shown to be phagostimulatory for the pea aphid *Acyrthosiphon pisum* Harris^53^. Melon aphid, however, didn’t encounter phagostimulatory response as it did not prefer either PRSV-infected or non-infected plants, despite these ratios being lower in non-infected plants and even more so in PRSV-infected plants. Significantly lower carbohydrate to amino acid ratios were also reported in CMV-infected plants^8^.

Showing both consistency and disparity with previous studies on NP viruses, PRSV modulated robust long-term effects on *A. gossypii* through enhanced nutritional quality of squash^5,15,35,54,55^. A thorough comparison of the present study with Blua *et al*.^9^ and Mauck *et al*.^8^ explains similarities and differences in aphid performance between these studies. The Blua *et al*. study^9^, as the current PRSV one, demonstrated that the longevity and intrinsic rate of increase of *A. gossypii* were significantly higher on ZYMV-infected vs. non-infected squash, mostly due to increased concentrations of individual amino acids. On the contrary, in Mauck *et al*. study^8^, the concentrations of essential amino acids, when compared with the respective non-infected squash plants, were significantly lower in CMV-infected plants. For instance, lysine and tryptophan were comparatively increased, while proline was significantly reduced in PRSV-infected plants. These two studies^8,9^ reported similar results to the present study on PRSV with regards to soluble carbohydrate concentrations. Blua *et al*. reported relatively higher total soluble carbohydrate concentrations whereas, Mauck *et al*.^8^ reported relatively higher and glucose and fructose concentrations in non-infected plants. Following the uptake of selectively manipulated amino acid and carbohydrate concentrations in PRSV-infected plants, the long-term effects of PRSV on *A. gossypii* appeared to be building up gradually during nymphal development and thrived later in the adulthood as evidenced by superior life history traits on PRSV-infected plants.

*B. tabaci* fecundity and developmental time showed no association with PRSV-induced changes in squash amino acids and sugars. This study showed disparity with recently published studies on NP^56^ and persistently-transmitted viruses^57,58^. In the NP virus study, *B. tabaci* was reported to preferably settle on non-infected chilli plants, but interestingly develop faster on CMV-infected chilli plants^56^. In the persistent virus studies, *Tomato spotted wilt virus* (TSWV) and *Pepper golden mosaic virus* (PEPGMV) offered fitness benefits to non-vector insects^57,58^. No effects in the present PRSV study could be due to intrinsic differences in nutrient uptake and metabolism and requirements between vector and non-vector insects. For instance, histidine and ornithine were reported to be the predominant amino acids determining *B. tabaci* development time on *Cucumis melo* L.^59^. No changes in *B. tabaci* development time in the present study could be due to low levels and lack of significant differences in these amino acids between infected and non-infected squash. Furthermore, *B. tabaci* has been shown to convert a large portion of the soluble phloem carbohydrates, predominantly sucrose, to the disaccharide, trehalulose –an insect haemolymph sugar composed of glucose and fructose^60^. Since sucrose concentrations have been equally minimal in both PRSV-infected and non-infected squash, the other predominant sugars in squash may have been nutritionally less relevant to whitefly physiology.

Deceptive signaling or attraction leading to short-term probing by vector on a NP virus infected plant and rapid dispersal thereafter is considered ideal for NP virus spread^2^. The probing behavior may or may not always be consistent with nutritional quality of plants, which appear to be increased, unaltered or reduced in NP virus infected plants^5,8^. Although these interactions appear to be context-specific, majority of NP viruses remain to be studied to draw any robust generic trends. Mauck and co-authors in their recent book chapter^7^ highlighted differences between *Potyviridae* (which have mostly neutral and positive effects on plant quality) and *Bromoviridae* (which have mostly neutral and negative effects on plant quality) in the NP viruses. The present study supports this observation, in that a strong positive relationship between a NP virus and vector in our PRSV pathosystem can be evidenced by increased vector performance through enriched amino acid and carbohydrate profiles of squash. Such association may appear epidemically less significant in a conventional sense, but may serve as an alternate virus dispersal strategy in nature as discussed recently by Carr *et al*.^6^. For instance, increased reproductive potential of *A. gossypii*, in the present study, could facilitate its population buildup on PRSV-infected plants in a short time. This population surge can increase the chances of the early development of alate *A. gossypii* due to crowding and nutrient exhaustion^61^. Therefore, colonizing insects such as *A. gossypii*, due to its virus-modulated fitness benefits, may be able to effectively promote virus spread to greater distances and form large inoculum foci^6^. This alternate strategy may be the reason why *A. gossypii* has been reported to be one of the most efficient PRSV vectors^32^. Furthermore, no PRSV-mediated effects on *B. tabaci* development and fecundity suggest the targeted manipulation of host plant quality by PRSV— more relevant to vector and less relevant to non-vector development.

### Concluding remarks

Our study demonstrated that PRSV selectively favors *A. gossypii*, but not *B. tabaci* fitness through host plant quality manipulation. The reduced emigration and increased fitness of *A. gossypii* on PRSV-infected plants can be attributed to increases in important amino acids: threonine, arginine, lysine, glycine, homocysteine and sugars: galactose, raffinose and cellobiose. Our findings shed light on a previously unexplored yet important pathosystem: PRSV-squash-*A. gossypii* and present a conclusive evidence of a strong positive correlation between a non-persistent virus and its vector. This relatively long-term favorable association between PRSV and *A. gossypii* could selectively favor virus spread. PRSV remains a chronic and severe threat to cucurbits in the southeastern US and elsewhere. The rapid *A. gossypii* population increase due to plant enrichment could lead to quicker dispersal and increased spatial spread of the virus. Increased alatae production could favor spread of virus farther and quicker. Perhaps, such spread is more conducive for a virus such as PRSV with a relatively restricted host range as opposed to viruses such as CMV with a broad host range. It is indeed obvious that additional NP virus pathosystems need to be investigated before any robust conclusions on virus-induced effects can be drawn, but this study has documented a previously unexplored pathosystem demonstrating virus-mediated selective manipulation of vector over non-vector insect via alteration in host plant quality.

## Material and Methods

### Host plants and PRSV inoculation

Two squash (cv. Goldstar) seeds per pot were planted using MiracleGro compost and grown in the greenhouse in whitefly-proof cages [47.5(*l*) × 47.5(*w*) × 93(*h*) cm] at 25 °C, 60% RH, and 16 L:8D photoperiod. Plants were thinned out one week after planting to retain one healthy seedling per pot. Two-week-old seedlings were mechanically inoculated with PRSV using 0.01 M phosphate inoculation buffer (pH 7). One-gram tissue of PRSV infected squash leaf was flash frozen with liquid nitrogen and mashed with 10 ml of 0.05 M phosphate buffer in a sterile mortar. The resulting inoculum was mixed with 0.1 g of each carborundum and Celite^**®**^ (Sigma Aldrich Inc., Darmstadt, Germany). Additional 0.1 g carborundum was dusted on a newly emerged leaf to induce abrasion. The sterile cheesecloth containing PRSV inoculum was rubbed lightly and spread over the entire leaf surface. Ten min following the inoculation, the leaves were lightly sprinkled with water to avoid excessive mechanical injury. Newly emerged leaves of inoculated plants showed intense mosaic pattern on the leaf lamina at two weeks post inoculation.

The infection status of PRSV-inoculated plants (*n* = 10) was confirmed using reverse transcription PCR. The total RNA from 100 mg symptomatic leaf tissue was extracted using RNeasy Plant Mini Kit (Qiagen Inc., USA). Initially, cDNA was synthesized from each RNA sample using GoScript® reverse transcription system (Promega Inc. Madison, USA). The cDNA was amplified by PCR using NIb1 (AGGTCATTTACAGCAGCCCC) and NIb2 (TCGAACTGGGAACCATCTGC) primers targeting the NIb gene. The PCR amplification involved 30 cycles at: 94 °C for 15 s, 53 °C for 15 s and 72 °C for 30 s and a final extension of 1 min at 72 °C. Gel-electrophoresis of PCR products revealed a 200 bp band in infected samples, with positive (PRSV-infected squash) and negative (non-infected recipient squash) controls. The non-infection status of non-infected squash plants (*n* = 10) from the same planting lot grown under same conditions was also confirmed using the same procedure.

### *A. gossypii* and *B. tabaci* settling bioassays

Settling bioassays were conducted following the modified procedure of Castle *et al*.^33^. One similarly-sized leaf from each of the PRSV-infected and non-infected squash plants was equidistantly placed in a settling arena. The arena consisted of a 15 cm plastic petri-plate platform, with a hole at the center, to which a 4(l) × 0.8(d) cm polypropylene tube was fixed. At the lower end of the tube, a 4(l) × 0.7(d) cm shell vial was attached. Each of the leaves remained attached to their respective plants throughout the experiment and was kept in position with Mylar film cages and cotton swabs. One hundred apterous adults of *A. gossypii* were starved for 1 h and placed in a vial. They could climb up through a polypropylene tube and arrive at the center with an equal access to either leaf. The entire arena was sealed to avoid the escape of aphids and was left undisturbed in the laboratory at 24 °C for 2 h. After 2hrs, the number of aphids settling on each of the treatment leaves was recorded. The experiment was replicated twice with 10 arenas with equal dimensions, and each arena represented a replicate (*n* = 20). New infected and non-infected replicate plants were used in each run, and the bioassay was conducted under laboratory conditions (25 °C; 12 h L:12 h D).

Just like *A. gossypii*, *B. tabaci* settling assay was conducted using PRSV-infected and non-infected squash. The arena to test *B. tabaci* settling was more simple and consisted of a plastic cylinder (150 mm diameter × 310 mm height) closed with a 15-cm diameter petriplate at the top^62^. The arena was sectioned at 9 cm from the top with two narrow slits (each 5-mm wide × 70 mm long) exactly opposite to each other. The same-sized leaves intact to the each PRSV-infected and non-infected plants were positioned inside the arena using a rubber foam strip (1.9 mm wide × 11.1 mm thick). One hundred non-viruliferous whiteflies maintained on cotton were aspirated into a 10-ml glass vial (VWR, Radnor, PA) and were released at the bottom of the arena. The number of whiteflies settling on each plant was recorded after 24 h. The experiment was replicated twice with 10 arenas set up each time (*n* = 20) with previously unused infected and non-infected replicate plants under laboratory conditions (25 °C; 12 h L:12 h D).

### *A. gossypii* emigration and immigration assays

No-choice emigration and immigration assays were conducted following the procedure of Eigenbrode *et al*.^1^, with slight modifications. The emigration arena consisted of a 25-cm flat wooden floor, on which fifty adult apterae, starved for 1 h, were released on a leaf from either a PRSV-infected or non-infected intact squash plant at one end and allowed to disperse to the other end. For immigration assays, 50 adult apterae starved for 1 h were released at the opposite end and allowed to immigrate towards a leaf from either PRSV-infected or non-infected intact squash plant. For both assays, numbers of emigrating and immigrating aphids were recorded and removed at every 10 min for 1 h. For each bioassay, experiment was repeated twice with 10 replicate plants (*n* = 20).

### *A. gossypii* and *B. tabaci* life history traits

A single randomly selected apterous *A. gossypii* adult was placed using a camelhair paintbrush on the underside of a leaf of the 6-week-old PSRV-infected and non-infected squash plants and confined with a clip cage. Each plant was fixed with two cages, and thirteen plants per treatment were used for a total of 26 replications per treatment. After 24 h of confinement, the females were removed, and only one neonate nymph was retained inside each cage. The life history of this single aphid was monitored throughout the entire life cycle. The nymphs laid by the subsequent generation adult were counted and removed daily. Fitness parameters such as nymphal period, adult period, pre-reproductive and reproductive periods, longevity, total fecundity and intrinsic rate of increase were estimated. The intrinsic rate of increase (*r*_*m*_) for each aphid was calculated using the equation of Wyatt & White^63^:

$${r}_{m}=0.747\frac{{lo}{{g}}_{e}{{\rm{N}}}_{d}}{{\rm{d}}}$$

where N_*d*_ is the number of nymphs produced during reproductive period, and “d” is the pre-reproductive period in days.

*B. tabaci* development time (egg-adult) and fecundity was studied on 6-week-old PRSV-infected and non-infected plants. Two clip cages each containing two adult whiteflies post-48h mating period were set up on each of ten PRSV-infected and non-infected plants. Whiteflies were removed after an oviposition period of 48 h and six randomly selected eggs per clip cage were retained. At least one surviving nymph per clip cage was monitored until adult emergence (egg-adult development). One randomly selected emerged adult per clip cage was moved to cotton plant soon after adult emergence and its fecundity was recorded.

### Soluble carbohydrate analysis

Between 8 and 15 mg of lyophilized leaf tissues from PRSV-infected and non-infected plants were used for the soluble carbohydrate analysis (*n* = 5). The analysis was carried out at the Complex Carbohydrate Research Center of University of Georgia. The dried samples were ground and suspended in 1 ml of water. The suspensions were vortexed briefly and filtered through glass wool. 500 μl of the sample suspension was used for the glycosyl composition analysis and the remaining was used for the High-Performance Anion-Exchange Chromatography coupled with Pulsed Electrochemical Detection (HPAEC-PAD) analysis. Glycosyl composition analysis was performed by combined gas chromatography/mass spectrometry (GC/MS) of the per-*O*-trimethylsilyl (TMS) derivatives of the monosaccharide methyl glycosides. These glycosides were produced from the sample by acidic methanolysis as described previously by Santander *et al*.^64^. This technique quantified the monosaccharides *viz*. ribose, arabinose, rhamnose, fucose, xylose and mannose in squash samples. The samples were analyzed by HPAEC-PAD using a Dionex ICS3000 system equipped with a gradient pump, an electrochemical detector, and an autosampler. The samples were separated using a Dionex CarboPac PA20 (3 × 150 mm) analytical column with an amino trap. The HPAEC-PAD analyzed mono- and di-saccharides such as galactose, glucose, fructose, raffinose and cellobiose.

### Free amino acid analysis

Free amino acids from PRSV-infected and non-infected plants were analyzed at the Molecular Structure Facility at the University of California, Davis (*n* = 5). Leaf tissue (150 mg) was flash frozen with liquid nitrogen and ground with sterilized mortar and pestle. The ground tissue was suspended in 600 μl of water: chloroform: methanol (3:5:12 v/v) extraction buffer. The resulting suspension was transferred to a 2 ml micro-centrifuge tube (Eppendorf North America, Hauppauge, NY) and was centrifuged at 14,000 rpm for 2 min. The supernatant was transferred to a new 2 ml micro-centrifuge tube. The pellet from centrifugation was re-suspended with 600 μl of extraction buffer and re-centrifuged as specified above. The 300 μl of chloroform and 450 μl of water was added to the combined supernatants. The suspension was centrifuged at 14,000 rpm for 2 min and the upper water: methanol phase was carefully transferred to a new 2 ml micro-centrifuge tube. The content was dried for about 3 h using a SpeedVac (Thermo Fisher Scientific, USA) and the resulting pellet was stored at −20 °C until further processing.

Samples were thawed and diluted with 100 nmol/ml S-2-Aminoethyl-L-cysteine (AE-Cys) diluent prior to the 50 µl injection. Free amino acids were separated using ion-exchange chromatography with a post-column ninhydrin (Wako Chemicals USA Inc.) reaction. Calibration of the amino acid analyzer Hitachi L8900 (Hitachi High-Technologies) was performed using amino acid standards (Sigma-Aldrich, St. Louis, MO). Absorbance was recorded at both 570 nm and 440 nm after the reaction with ninhydrin to determine the response factor for each individual amino acid and to quantify levels relative to the known amino acid standards. The included internal standard (AE-Cys) was used to correct for any variances in injection volume due to the auto-sampler.

### Statistical analyses

Data analyses were performed in R version 3.3.0 (The R Foundation for Statistical Computing). Differences in (i) number of aphids and whiteflies preferring either non-infected or PRSV-infected leaves, and (ii) melon aphid fecundity and intrinsic rate and whitefly fecundity on non-infected and PRSV-infected plants were analyzed using single factor ANOVA and mean separation with Tukey’s HSD posthoc test (‘aov’ and ‘TukeyHSD’ functions in R). Differences in (i) the median development times of melon aphid (NP, AP, longevity, RP) and whitefly (egg-adult) and (ii) concentrations of soluble carbohydrates and free amino acids between non-infected and PRSV-infected plants were analyzed using Mann-Whitney *U* Test (‘wilcox.test’ in R). A non-linear relationship between number of emigrating and immigrating melon aphids (response variables) and treatment and time (explanatory) variables was tested using generalized linear model procedure (GLM procedure, ‘nlme’ and ‘lme4’ libraries in R). The procedure tested if the number of aphids were significantly different between non-infected and PRSV-infected plants across different time points. A model selection, to determine the better of two GLM models, was performed using Akaike Information Criterion (AIC) values^65^. The data were analyzed with a Poisson distribution and a log link mode. The repeated measures ‘Anova’ function from the ‘car’ package in R was used to report Chi-squared and p-values for treatment, time and interaction effects.

## Data Availability

The datasets generated and analysed during the current study are available from the corresponding author on reasonable request.
